# Food Behavior in Emergency Time: Wild Plant Use for Human Nutrition during the Conflict in Syria

**DOI:** 10.3390/foods11020177

**Published:** 2022-01-10

**Authors:** Naji Sulaiman, Andrea Pieroni, Renata Sõukand, Zbynek Polesny

**Affiliations:** 1 Department of Crop Sciences and Agroforestry, Faculty of Tropical AgriSciences, Czech University of Life Sciences Prague, Kamýcká 129, 16500 Praha-Suchdol, Czech Republic; sulaimann@ftz.czu.cz; 2 University of Gastronomic Sciences, Piazza Vittorio Emanuele II, 9, 12042 Pollenzo, Italy; a.pieroni@unisg.it; 3 Medical Analysis Department, Faculty of Science, Tishk International University, Erbil 44001, Iraq; 4 Department of Environmental Sciences, Informatics and Statistics, Ca’ Foscari University of Venice, Via Torino 155, 30174 Venezia, Italy; renata.soukand@unive.it

**Keywords:** Eastern Mediterranean, emergency human behavior, ethnobotany, *Sleeq*, traditional food, *Zaatar*

## Abstract

Wild food plants (WFPs) have been an important source of human nutrition since ancient times, and it particularly revives when conventional food is not available due to emergency situations, such as natural disasters and conflicts. The war in Syria has entered 10 years since it started in 2011, and it has caused the largest war-related crises since World War II. Nearly 60% of the Syrian population (12.4 million people) are food-insecure. WFPs are already culturally important in the region, and may be supplementing local diets during this conflict. Our study aimed to uncover the conflict’s effect on the use of WFPs and to know what species are consumed by local people during the current crisis. The fieldwork was carried out between March 2020 and March 2021 in the Tartus governorate located in the coastal region of Syria. Semi-structured interviews were conducted with 50 participants (26 women and 24 men) distributed in 26 villages along the study area. We recorded the vernacular names, uses, plant parts used, modes of preparation and consumption, change in WFP use before and during the conflict, and informants’ perceptions towards WFPs. We documented 75 wild food plant species used for food and drink. Almost two-thirds (64%) of informants reported an increase in their reliance on wild plants as a food source during the conflict. The species of *Origanum syriacum*, *Rhus coriaria*, *Eryngium creticum*, and *Cichorium intybus* were among the most quoted species by informants. *Sleeq* (steamed leafy vegetables), *Zaatar* (breakfast/dinner food), and *Louf* (soup) were the most popular wild plant-based dishes.

## 1. Introduction

Food is located at the bottom of Maslow’s hierarchy of human needs, where any threat to these basic needs could impact human behavior and bring emergency reactions [1]. Despite the importance of food, hunger remains a main part of human history, as well as the present. According to FAO [2] estimations, the number of hungry people will exceed 840 million by 2030. On the other hand, two billion people (25.9% of the global population) experienced hunger or did not have regular access to nutritious and sufficient food in 2019. Much of the recent increase in food insecurity can be attributed to the greater number of conflicts and climate change impacts [2]. In general, wartime is characterized by deterioration of life quality, as wars often lead to mass human migrations, armed-conflict zones, and economic crises. Conflict areas lack basic living necessities, such as appropriate shelter, drinking water, and medicine. Furthermore, sufficient and diverse food sources are scarce, while hunger and nutritional deficiency-related diseases are widespread [3].

Since ethnobotany has been described as the science of survival [4], its importance increases during armed conflicts. In crises where conventional food is not available, people turn to their traditional ecological knowledge to secure their needs from the surrounding environment, where wild plants serve as the main food source. Generally, the use of wild plants as an emergency food supply has been present throughout the entire history of humankind [5]; gathered plants from the wild have been used to cope with food shortages in many regions around the globe [6,7], as well as to alleviate poverty levels [8]. Several studies have highlighted wild plant use and its importance for human survival in times of conflicts, particularly in Bosnia and Herzegovina during the Balkan War [3,9,10], as well as in the Netherlands with the food famine during World War II [11].

In the last two decades, the world experienced an increase in its number of conflicts. Only between 2000 and 2014, there was an average of 35 active conflicts every year around the globe [12]. Since the so-called “Arabic Spring” started in the Middle East a decade ago, many countries have fallen into civil wars, local conflicts, and economic crises. In March 2011, Syria entered an armed conflict that caused the largest displacement crisis since World War II. So far, 6.2 million Syrians are internally displaced, and 5.7 million are registered as refugees outside of the country. Soaring food and fuel prices, stagnant salaries, loss of livelihoods, and reduced food production have led to widespread food insecurity across Syria [13]. Nearly 60% of the Syrian population (12.4 million people) are food-insecure [14]. FAO [15] reported that wheat production is at less than 25% of pre-conflict levels, which has significantly affected food security in the country. Recent statistics from Syria show low dietary intake of several key foods and nutrients compared to the minimum recommended level, such as vegetables, which is at 25% less than the global level and almost 50% less than the theoretical minimum-risk exposure level [16]. The economic conditions resulting from the ongoing conflict are not only affecting the war battle areas, but being reflected across the country. 

In Syria, as a part of the Mediterranean basin, cooked vegetables and salads made from wild greens have been particularly important as local traditional foods since ancient times [17]. However, wild food plants (WFPs) have not been widely studied in the Syrian context [18,19], and while teas prepared from wild plants have been marginally covered [20,21,22], there are no studies about the use of wild plants with a focus on the effects of the conflict. On the other hand, studies from surrounding countries, such as Lebanon [23,24], Cyprus [25], Iraqi-Kurdistan [26], Palestine [27], and Turkey [28] clearly show the importance of wild plants in the food culture of the East Mediterranean region. Similarly, the gathered plants from the wild have been a part of the Syrian local cuisine before the conflict and its associated economic crisis [19]. However, we hypothesize that the use of WFPs has changed during the ongoing conflict. Hence, the overall interest of our study was to uncover the conflict’s effect on WFP use and to know what species are consumed by local people during the current crisis. We particularly aimed to conduct an inventory of the wild plant species used as food in the region, and to document the ethnobotanical knowledge, including modes of preparation and consumption, as well as to highlight the perceptions of local people toward use of WFPs both before and during the conflict. We also aimed to summarize the nutritional benefits of the most-reported WFPs.

## 2. Materials and Methods

### 2.1. Study Area

**Figure 1:**
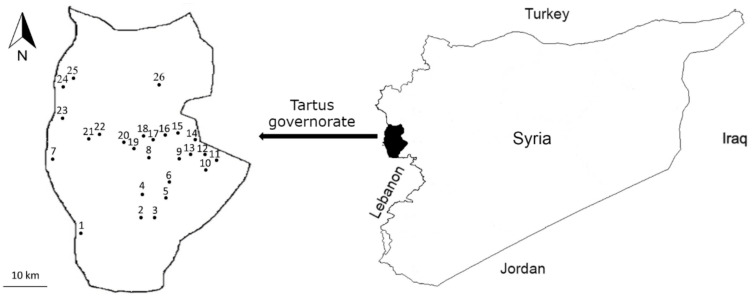
Study area map, the Tartus governorate and studied villages: 1. Al-Hamidiyah, 2. Ain Dabesh, 3. Al-Mitras, 4. Al-Sawma’a, 5. Bishrael, 6. Sibbeh, 7. Tartus, 8. Draykish, 9. Bait Yousef, 10. Husn Suleiman, 11. Annabi Saleh, 12. Annabi Matta, 13. Bestan Assouj, 14. Ain Dlaimah, 15. Fajlit, 16. Al-Tuffaha, 17. Kafr Tallesh, 18. Krafes, 19. Al-A’ujah, 20. Kawkab, 21. Bazughah, 22. Brmmanet Raad, 23. Maten Al-Sahel, 24. Al-Rawda, 25. Dahr Safra, 26. Sourani.

The study was conducted in the Tartus governorate (Figure 1), one of the 14 governorates of Syria. Initially, we aimed to include more than one governorate as a study area, but due to COVID-19 restrictions, the first author could not move beyond the Tartus Governorate. Tartus (34.9° N and 35.9° E) is bordered by Lebanon in the south, Syrian governorates of Latakia in the north, and Homs and Hama in the east. Tartus forms roughly half of the Syrian Mediterranean coastline, extending to 183 km [29]. The governorate occupies a territory of 1896 km^2^ and has a population of 1,114,000 inhabitants [30]. The climate is Mediterranean, and characterized by hot and dry summers, moderate in the mountains, whereas winters are mild and wet. Average annual rainfall varies from 800 to 1000 mm [31]. The region has a long history that dates back to the era of Phoenicians who built several cities in the area, such as Arwad and Amrit. Arwad, the only inhabited island in the Eastern Mediterranean coastline, has been continuously occupied since at least the third millennium BC [32]. Tartus is a multi-ethnic and religious region consisting of Alawites, Sunnis, Ismailis, and Christians, with percentages of 69%, 18%, 7%, and 6%, respectively. Arabs are the majority in the governorate, with Greek and Turkmen minorities [33]. 

So far, the region has been relatively quiet and safe during the Syrian conflict. Tartus is entirely under the government control; however, it witnessed several security incidents [34]. It is facile to observe the lack of young men, since most of them were requested to join or taken to the Syrian army as a result of the ongoing war in other parts of the country. The unfinished conflict did not yet reveal the exact numbers of casualties and wounded young men of the governorate, which could exceed tens of thousands according to the local people. Tartus houses more than 169,000 displaced people from other Syrian governorates [35]. Although the study area is not significantly affected by war battles as other regions in Syria, the community is suffering from the side-effects of war, such as food shortage, economic sanctions, limited affordability of the market products, labour shortage, fuel insufficiency, frequent power outages, and general lack of agricultural production input [36,37]. Similarly to other governorates in Syria, around 50% of households in the Tartus governorate had inadequate food consumption in the second half of 2020 [38].

### 2.2. Fieldwork, Data Collection and Data Analysis

The study was conducted between March 2020 and March 2021. The participants were chosen using a combination of purposive and convenient sampling methods [39,40]. We asked people in the village’s streets if they would agree to be interviewed; the basic criteria for an informant to be selected was that he/she was a breadwinner and usually used wild plants for food or beverage. Semi-structured interviews were conducted with 50 participants (26 women and 24 men), aged between 25 and 97 years. Participants were chosen from 26 villages along the study area to include members from all religious and ethnic groups in the study. 

Informants were asked to list all the wild plants they used for food and beverage preparation, their vernacular names, the parts used, and mode of preparation and consumption. Respondents were asked to report any changes in their use of WFPs prior to and during the conflict. Perceptions of informants towards WFPs were documented using open-ended questions, such as: Why do you use WFPs? Why were you using WFPs before the conflict started?

Verbal consent was always obtained before each interview, and the Code of Ethics of the International Society of Ethnobiology was followed [41]. A research permit was obtained from the General Commission for Scientific Agricultural Research and Tartus municipality (number: 9893; date: 5 April 2020). All interviews were conducted in the Arabic language. Plants were identified with the help of local taxonomists and the herbarium staff of the American University of Beirut, while nomenclature followed World Flora Online [42]. The collected voucher specimens were deposited in the Herbarium of the American University of Beirut (BEI). When the plant sample was not available, identification was done based on a detailed description of the plant and its habitat, as well as the local name(s) provided by the informants. Taxa that were not capable of being classified up to the species level were identified at the genus level. All local plant names were transcribed from the recorded local languages using the Latin alphabet.

Collected data were compared with previously published ethnobotanical studies from the Eastern Mediterranean region and adjacent countries [18,19,23,24,25,28,43], in order to determine differences and similarities in WFPs’ uses. Data on the nutritional benefits of most reported species were obtained by reviewing the literature available online [44,45,46,47,48,49,50,51,52,53,54]. The relative importance of the reported plants was obtained by calculating the relative frequency of citation (RFC) for each species by dividing the number of informants mentioning the plant by the total number of informants. The RFC value ranged from 0 to 1, with 0 as a theoretical value indicating that no informant mentioned the species, and 1 as the highest value indicating that all informants mentioned it [55].

## 3. Results

### 3.1. Pre-Conflict vs. During-Conflict Use of Wild Food Plants

The majority of our study respondents (94%) used WFPs before the conflict, whereas 6% of them started to use those plants only after the conflict started a decade ago. Informants showed that WFPs have always been a part of their traditional diet. However, such meals served as complementary food before the conflict, while they became among one the main dishes in recent years. Almost two-thirds (64%) of informants reported an increase in their reliance on wild plants as a source of food during the conflict compared to the pre-conflict time. On the other hand, 34% of the informants reported a no-change status in their reliance, whereas 2% stated a decrease. The main reported reason behind the apparent increase of reliance on WFPs was the economic crisis that hit the whole country after the war, which affected the food prices and purchase power, while the reason for the minor percentage of decrease in reliance was the move from a rural to urban area. The conflict affected accessibility and WFP species selection; informants reported that during the conflict, they started to consume wild plant species that grew in anthropic sites and their surrounding orchards, such as *Eryngium creticum* and *Malva sylvestris*. On the other hand, species that grew in high mountains and remote areas, such as *Gundelia tournefortii*, witnessed a decrease in use during conflict years due to obstacles regarding movement and security concerns. According to our informants’ statements, traditional knowledge of WFPs was noticeably decreasing during the stable economic situation in pre-conflict years. However, it was revived after becoming necessary to secure food during the conflict. 

### 3.2. Perceptions of Local People towards Wild Food Plants Pre and during the Conflict

Informants’ statements show a clear difference in perceptions and reasons for using WFPs during and before the conflict. The main motives behind the use of WFPs in the pre-conflict era were represented by the tendency to eat healthy and organic food, efforts to diversify the food and taste, and enjoyment of the activity of gathering plants from the wild. On the other hand, the difficulty to afford the market food products was the main reason for WFP use during the conflict. The majority of informants (58%) reported that the natural ecosystems helped to substitute market products through the gathered plants, and consequently, to save money. The mutual perception among the local people was that some of the young generation’s views on WFPs did not change during the conflict compared to the pre-conflict time. These young people, especially those who did not bear the responsibility of securing food for the household, perceived WFPs as non-prestigious food and felt ashamed to share information related to this food with their peers.

### 3.3. Diversity of the Wild Food Plants

Our study documented 75 wild plant species used for food and beverage preparation by the local people. One taxon was identified only down to the genus level. The total of 75 taxa belonged to 70 genera and 28 botanical families. The most-represented families were Compositae (15 species), Fabaceae (8 species), Lamiaceae (7 species), Apiaceae, and Rosaceae (5 species each). Compositae included plants that are mostly prepared as cooked vegetables. On the other hand, Lamiaceae mainly included the spices and herbs that are consumed, dried, and ground, whereas Rosaceae included the species that are consumed mostly as fresh fruits. Species mentioned only once or twice in the survey were excluded from further analysis.

Sixty-four plant species were reported by at least 6% of informants (Table 1). The following species showed a level of quotation above 80% of respondents (in descending order of quotation): *Origanum syriacum*, *Rhus coriaria*, *Eryngium creticum*, *Cichorium intybus*, *Micromeria myrtifolia*, *Allium ampeloprasum*, *Cirsium vulgare*, *Gundelia tournefortii*, *Scandix pecten-veneris*, *Malva sylvestris*, *Anchusa strigosa.* On the other hand, the species *Nasturtium officinale*, *Rumex acetosa*, *Thymus vulgaris*, and *Arum maculatum* were used by 60–80% of informants. 

### 3.4. Most Common Wild Plant-Based Dishes

#### 3.4.1. Sleeq

*Sleeq* is the most popular wild plant-based dish in the study area. It is prepared from gathered wild leafy vegetables. The name *Sleeq* is probably derived from the word *Saleeq,* which in the Arabic language means boiled food; however, the dish is mainly prepared by steaming, rather than boiling. The same dish was also called *Mhabbleh* by some informants, which locally refers to steamed food. There is no limitation to what kind of or how many wild vegetables can be included in the preparation. The most common species used in the preparation of *Sleeq* are *Scandix pecten-veneris*, *Cirsium vulgare*, *Allium ampeloprasum*, *Erodium acaule*, *Cichorium intybus*, *Anchusa strigosa*, *Gundelia tournefortii*, *Silybum marianum*, *Rumex acetosa*, *Anemone coronaria*, *Malva sylvestris,* and *Urtica dioica*. Practically every wild leafy vegetable that is able to be gathered by the collectors can be included in the dish, since it is sometimes difficult to gather a sufficient amount from one species only. The young shoots are the main part used in the *Sleeq*. The dish is mostly prepared in a local traditional clay pan called *Meqli* (see the video abstract), where chopped onion is fried in olive oil, then cut wild leafy vegetables are added. Some informants add a small amount of chickpea and/or softly ground bulgur if available. Several informants reported that it is possible to use *Sleeq* as a topping on the dough to be prepared as pizza, with the same mentioned receipt excluding chickpea and bulgur. *Sleeq* is a seasonal food connected with the availability of wild leafy vegetables usually collected from January to April.

#### 3.4.2. Zaatar

Traditional food in many regions in Syria. It is a sour spicy dish consumed by mixing with olive oil, usually in breakfast and dinner meals, as well as in *Zaatar* bread/pizza. The dried and ground leaves of *Origanum syriacum*, the main ingredient of *Zaatar*, are mixed with dried ground fruits of *Rhus coriaria,* and roasted seeds of *Sesamum* spp. in the following proportion: 1.0 of *Origanum syriacum*, 0.75 of *Rhus coriaria*, 1.0 of *Sesamum* spp. Dried fruits of *Pistacia terebinthus/Pistacia atlantica* and seeds of *Foeniculum vulgare* may be sprinkled as spices. *Zaatar* is usually stored dried in jars or plastic bags, and consumed over the whole year.

#### 3.4.3. Louf Soup

*Louf* is a traditional soup consumed only in some parts of the Syrian coastal mountains, especially by the Alawite and Ismaili cultural-religious groups. The soup has a sour-astringent taste with a thick texture. *Arum maculatum* is the main ingredient in *Louf* soup; its dark-green leaves are loosely cut and steamed for around half an hour. Afterwards, olive oil and bulgur are added, then the water of boiled *Rhus coriaria* is added after disposing of *Rhus coriaria. Arum maculatum* contains a high amount of calcium oxalate, which is a toxic compound [56]; however, as local people are aware of this toxicity, it is thus prepared by boiling for more than an hour to detoxify it. The dish is characterized as a winter soup, as *Arum maculatum* is usually available from December to April. Some informants reported that they store it in glass jars in the refrigerator and consume it throughout the year.

#### 3.4.4. Shabshuleh

**Figure 2:**
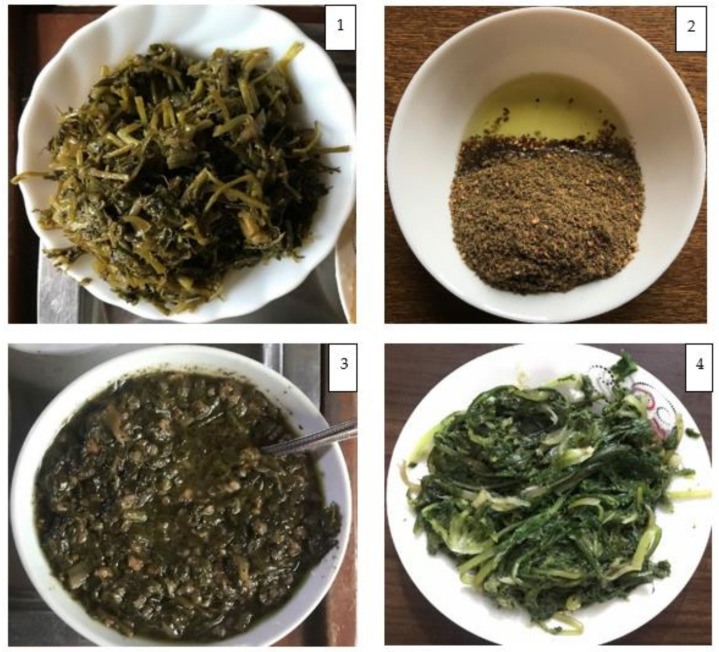
The most popular wild plant-based dishes. (**1**): *Sleeq* dish; (**2**): *Zaatar* dish with olive oil; (**3**): *Louf* soup; (**4**): *Shabshuleh* dish.

The young aerial part of *Cichorium intybus* is boiled for around half an hour, then olive oil, crushed garlic, and a little bit of lemon juice are added (Figure 2). It is typically consumed between January and April before *Cichorium intybus* starts flowering.

## 4. Discussion

### 4.1. Emergency Behavioral Reaction: Reliance Increase on Wild Food Plants during the Conflict

The reported increase of wild plant use during the conflict compared to the pre-conflict time clearly shows the effect of the conflict-associated economic crisis on the informants’ behavior toward food-securing. This finding is strongly supported by the respondents’ statements that highlighted economic reasons as the main motive behind such an increase. Wild plant foraging shifted from a complementary and entertainment activity for many informants a decade ago, to an important and necessary action in recent years. Such an emergency reaction went beyond being individual in other countries; in the winter of 1944–1945, the Dutch government provided information on wild plants and other famine food sources in the so-called “wartime cookbooks” [11]. Similarly, Redžić [3] broadcasted information over the radio to assist local people to find edible plants as a quick reaction to food scarcity during the siege of Sarajevo in the Balkan war. Several other studies around the globe concluded that wild plant use and their related traditional knowledge served as a coping mechanism in response to food shortages [57,58,59]. Hence, traditional ecological knowledge (TEK) has to be conserved by societies, apart from its cultural importance, due to its ability to save the lives of millions of people in times of future crisis. Many obstacles stand in the way of TEK preservation, such as the invasion of fast food and prestige-related perception of local food. However, several strategies could be followed to preserve TEK, such as marketing the local food based on traditional knowledge, documenting TEK, raising awareness of its importance, and teaching the skill of recognizing and collecting wild plants to school students. 

Changes in diet in the study area have mainly been represented by the decrease in buying market food products and increase in the use of WFPs, particularly leafy vegetables. Most commodities’ prices in Syria have increased tens of times since the beginning of the conflict. However, a few products are still affordable by the majority due to the government supporting these products. Bread, which is the main part of every meal, is one of these affordable commodities at 0.06 US Dollars per 1 kg (1 USD = 3500 Syrian Pounds according to the market exchange rate of late October 2021). Olive oil is produced by the majority of people in the study area where olive orchards dominate the landscape. Vegetable prices in the market vary between 0.3 to 1.4 USD for 1 kg, and prices for 1 kg of meat range between 2.9 and 5.7 USD. Those prices are considered relatively high and commonly unaffordable compared to the average monthly salary of 149,000 SP ≈ 43 USD [60]. Hence, we find that bread, olive oil, and gathered WFPs form the key ingredients for the most affordable food (e.g., *Sleeq*). Wild vegetables are also sold in the local markets at a lower price than other cultivated vegetables; for instance, the price of 1 kg of wild leafy vegetables (*Sleeq*) varies between 0,28 and 0,42 USD. We observed that several informants attempted to cultivate some wild species (e.g., *Origanum syriacum*, *Thymus vulgaris*) in their home gardens. Some people living in the cities and town centres (where access to wild plants is limited) depended on their relatives and friends in rural areas to gather WFPs for them.

### 4.2. Comparison of the Reported Wild Plant Diversity with Other Regions and Cultural Importance of Some Reported Species

**Figure 3:**
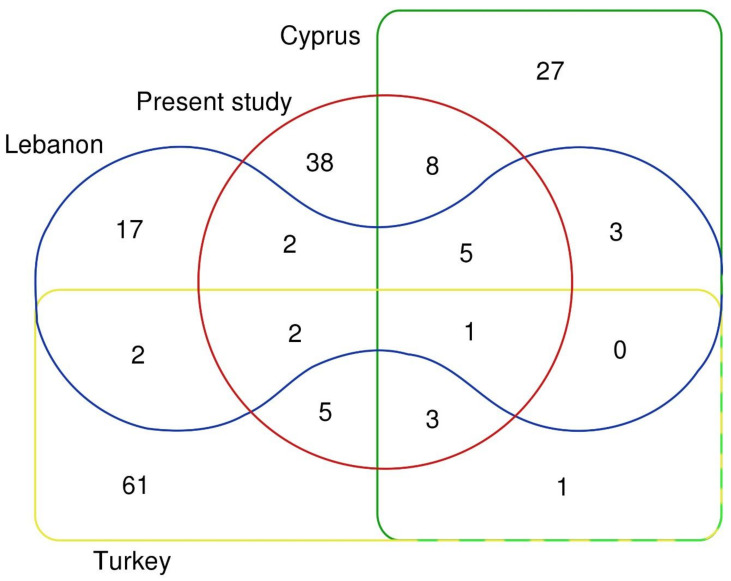
Overlap of used wild food plants between our study and selected studies from some East Mediterranean countries (numbers refer to the overlapping species).

The total number of 75 documented WFPs in the study area represents relatively high diversity compared to the Eastern Mediterranean region. It demonstrates a richness in the wild-related food culture. Kawas et al. [18] documented eight wild edible species out of 145 wild plants found in Hama Steppe in central Syria. On the other hand, 42 wild plant species are used in the Assyrian cuisine in the Eastern Syrian-Turkish borderland [19]. The majority of the reported plants in our study were not mentioned in either study of Kawas et al. [18] or Abdalla [19]; this is possibly due to the difference in ecosystems and vegetation types. Lower diversity of WFPs was found in Lebanon with 32 recorded species [23,24]. On the other hand, high diversity was noted in Adana in Turkey (76 documented species) and Cyprus with 78 recorded species [25,28]. On the other side of the Syrian borders, Pieroni et al. [43] documented 34 taxa used as WFPs in the Kurdistan region in Northern Iraq. The high diversity in species used in our study area could be attributed to the emergency situation, which pushed local people to use every edible plant available in the surrounding environment. Figure 3 shows the overlap in the used WFPs between our study (64 species in Table 1), and studies selected from the closest countries to our study area represented by Lebanon [23], Cyprus [25], and Turkey [28]. The overlap demonstrates the Mediterranean influence on the wild plant-based diet in our study area, where 41% of our species (26 out of 64) was used in food preparation in the other compared studies. More than one-fourth (17) of our reported species were used in Cyprus, and some species were similarly prepared; this is possibly due to the geographical closeness to our study area. In addition to the overlap in used species between the selected study areas, more similarities were found in genera; this is due to the presence of different species that belong to the same genus. The use of *Gundelia tournefortii* is documented in all compared studies; this is possibly due to its pleasant taste, as our study informants reported that it is one of the most preferred WFPs. It is usually steamed in olive oil with minced meat or chickpeas. Several plant species were found to be unique to the study area, such as *Arum maculatum,* which is a very popular and traditional soup (*Louf*) in some parts of the study area. All the species that comprise the most common wild plant-based dishes are culturally important and often frequently used. Despite that some of these species are less available nowadays in the wild, they still remain highly preferred, and this is possibly due to their pleasant taste or their importance in the local culture as a main part of the traditional diet. Based on the results and our observation, the sour-astringent taste was found to be preferred by many informants, especially from Alawites and Ismailis, which was demonstrated by the high use of some wild species, such as *Arum maculatum* and *Rhus coriaria,* which were described by some informants as “the sour of Phoenicians”.

### 4.3. Nutritive Value of the Most Quested Species

There is a deficiency of data on nutritional status in Syria, especially considering that the situation is getting worse every year with the continued conflict, economic sanctions, and conflict-related chaos and corruption. However, a report from WFP [61] showed a high level (12.7%) of chronic malnutrition amongst children under the age of five. Anaemia is widespread amongst both children under the age of five and women with a prevalence of 25.9% and 24.5%, respectively. Here, iron-rich WFPs, such as *Scandix pecten-veneris*, *Gundelia tournefortii*, and *Nasturtium officinale*, as well as other dark-green leafy wild vegetables, such as *Arum maculatum*) could play a significant role in preventing anaemia (Table 2). In times of COVID-19 and immunity-related diseases, species rich in zinc content, such as *Scandix pecten-veneris* and *Allium ampeloprasum,* could significantly help local people due to the direct effect of Zn on the overall activity of the immunity system [62]. Some of the reported species, especially those which are quoted by the majority, have shown a general richness in nutritional value and health benefits (Table 2). Species such as *Origanum syriacum* and *Thymus vulgaris* demonstrated a richness in antioxidants. The protein content was particularly high in some species, such as *Rumex acetosa*. Hence, for instance, the wild plant-based meal of *Sleeq* which contains wild leafy vegetables (rich in minerals and vitamins), olive oil (a significant source of unsaturated fatty acids), bulgur (protein-rich cereal), and bread (a main source of carbohydrates) serves as a good source of essential nutrients for the human body. 

## 5. Conclusions

The field study that we conducted among the local people in the coastal region of Syria showed a remarkable level of reliance on WFPs as a source of human nutrition. The study demonstrated the increased use of WFPs during the conflict compared to the pre-conflict time. Our results strengthened the findings of previous studies that ethnobotanical knowledge functions as a coping method for food shortage. We documented 75 wild plant species used in food and beverage preparation with a relatively high diversity comparing to other studies from the Eastern Mediterranean. The most common wild food-based dishes and their preparation mode were documented. Some species, such as *O. syriacum*, *G. tournefortii,* and *R. acetosa* demonstrated a richness in nutrient content. More research will help determine the exact nutritional role that these WFPs play in supplementing local diets during the conflict. Understanding the perceptions of local people towards WFPs could help in planning successful promotion of some nutritive species. Furthermore, future studies should consider the sustainability of WFP use and how these plants could be protected during crises.

## Figures and Tables

**Table 1:** Wild plant species used in food and beverage preparation in the study area.

Botanical Family	Latin Name (Voucher Specimen Code)	Local Name(s)	Part Used	Mode of Preparation and Consumption	Relative Frequency of Citation (N ^1^ = 50)
Amaranthaceae	*Amaranthus retroflexus* L. (NC ^2^)	*Qttaifeh*	Young aerial part	Steamed with other WFPs (*Sleeq*); fried with eggs.	0.16
	*Beta vulgaris* L. (NC)	*Selq* *barri*	Young aerial part	Boiled	0.1
	*Chenopodium bonus-henricus* L. (NC)	*Sabanekh barri*	Leaves	Boiled	0.08
Amaryllidaceae	*Allium ampeloprasum* L. (Sulaiman 27)	*Kerrat,* *Twaimeh*	Young aerial part and bulb	Fresh; steamed with *Sleeq*; fried with eggs, or with minced meat.	0.96
Anacardiaceae	*Pistacia atlantica* Desf. (NC)	*Betem*	Leaves and fruits	Spice: leaves are added specifically to the traditional soup *Qishq* (it also serves as a preserver); fruits are added to *Zaatar*.	0.36
	*Pistacia terebinthus* L. (Sulaiman 3)	*Betem*	Leaves and fruits	Spice: leaves are added specifically to the traditional soup *Qishq* (it also serves as a preserver); fruits are added to *Zaatar*.	0.36
	*Rhus coriaria* L. (Sulaiman 14)	*Summaq*	Fruits	Spice added to: salads, *Zaatar*, boiled potato, and to the traditional soup *Louf* (*A.* *maculatum)*	0.98
Apiaceae	*Ammi majus* L.	*Khelleh*	Inflorescence	Added to yerba mate (to substitute an amount of yerba mate) [20].	0.06
	*Apium nodiflorum* (L.) Lag. (NC)	*Qarrah*	Young aerial part	Appetizer	0.44
	*Eryngium creticum* Lam. (Sulaiman 11)	*Qers* *anneh*	Young aerial part	Salad; steamed with *Sleeq*.	0.98
	*Foeniculum vulgare* Mill. (Sulaiman 37)	*Shamra*	Aerial part	Spice added to *Zaatar* and soups; fried with: eggs/ meat/potato or *A. ampeloprasum*.	0.4
	*Scandix pecten-veneris* L. (Sulaiman 20)	*Hert* *manneh*	Young aerial part	Steamed with *Sleeq*.	0.84
Araceae	*Arum maculatum* L. (Sulaiman 31)	*Louf*	Young aerial part	Soup: boiled with *R. acetosa, R. coriaria*, bulgur (cracked, parboiled groats of *Triticum durum* Desf.), and olive oil.	0.66
Asparagaceae	*Asparagus acutifolius* L. (Sulaiman 30)	*Halyoun*	Shoots	Fried with eggs; salads; steam frying.	0.58
Boraginaceae	*Anchusa strigosa* Banks and Sol. (Sulaiman 46)	*Balasoun*	Young aerial part and underground stem	Fried; steamed with olive oil, and garlic or onion.	0.82
Brassicaceae	*Lepidium ruderale* L. (NC)	*Reshad* *barri*	Young aerial part	Appetizer	0.48
	*Nasturtium officinale* R.Br. (NC)	*Jarjeer*	Young aerial part	Appetizer	0.78
	*Sinapis arvensis* L. (Sulaiman 18)	*Fejjaileh*	Young aerial part	Steamed with *Sleeq*; boiled, and then olive oil and lemon are added; fried with eggs.	0.22
Campanulaceae	*Michauxia campanuloides* L’Hér. (NC)	*Qarf* *awn*	Young aerial part, root	Steamed with *Sleeq*; steamed with onion and carrot.	0.16
Caryophyllaceae	*Silene dioica* (L.) Clairv. (Sulaiman 32)	*Lbas* *alqetah*	Young aerial part	Steamed with *Sleeq.*	0.2
Compositae	*Centaurea calcitrapa* L. (Sulaiman 4)	*Qellaibeh,* *Dardar*	Young aerial part	Steamed with *Sleeq*.	0.18
	*Cichorium intybus* L. (Sulaiman 43)	*Hendbeh*	Young aerial part	*Shabshuleh*: boiled, then olive oil and lemon juice and garlic are added; steamed with *Sleeq*.	0.98
	*Cirsium vulgare* (Savi) Ten. (Sulaiman 8)	*Qessitah*	Leaves midrib and underground stem	Steamed with *Sleeq*; steamed with chickpea.	0.88
	*Crepis sancta* (L.) Bornm. (NC)	*Del* *’a* *alhelou,* *Qers* *alhelou*	Young aerial part	Steamed with *Sleeq*.	0.08
	*Crepis vesicaria* L. (Sulaiman 45)	*Harbsees*	Young aerial part	Steamed with *Sleeq*.	0.36
	*Cynara syriaca* Boiss. (NC)	*Ardi* *shouki barri,* *Kharshouf*	Inflorescence	Steamed with minced meat.	0.1
	*Gundelia tournefortii* L. (Sulaiman 19)	*Salbeen*	Leaves midrib and underground stem	Steamed with chickpea and olive oil; steamed with onion and olive oil; steamed with minced meat; cooked with rice.	0.86
	*Helminthotheca echioides* (L.) Holub (Sulaiman 44)	*Khishan*	Young aerial part	Steamed with *Sleeq*.	0.28
	*Leontodon hispidus* L. (Sulaiman 16)	*Sliq* *aloud*	Young aerial part	Steamed with *Sleeq*.	0.28
	*Matricaria chamomilla* L. (NC)	*Babounej*	Flowers	Tea; added to yerba mate.	0.08
	*Notobasis syriaca* (L.) Cass. (NC)	*Shok* *aljamal,* *Kherfesh,* *Qailouh,* *Shouk* *alqed*	Leaves midrib	Steamed with onion and olive oil; boiled then steamed with minced meat.	0.12
	*Silybum marianum* (L.) Gaertn. (Sulaiman 48)	*Labboun*	Leaves midrib and underground stem	Steamed with onion and olive oil; steamed with *Sleeq*; steamed with chickpea and olive oil, steamed with minced meat and then mixed with yogurt.	0.34
	*Sonchus oleraceus* (L.) L. (NC)	*Khesaiseh, Asat alraa’I, Elk alghazal*	Young aerial part	Steamed with *Sleeq.*	0.18
	*Tragopogon pratensis* L. (Sulaiman 9)	*Daqen* *alshaikh,* *Daqen* *alkhouri*	Young aerial part	Steamed with *Sleeq*.	0.1
Cucurbitaceae	*Bryonia cretica* L. (Sulaiman 36)	*Atairisheh*	Leaves	Fried with eggs.	0.36
Geraniaceae	*Erodium acaule* (L.) Bech. and Thell. (Sulaiman 10)	*Mssaikeh*	Young aerial part	Steamed with *Sleeq*.	0.48
Lamiaceae	*Mentha piperita* L. (NC)	*Nana* *’a* *barri*	Leaves and shoots	Appetizer; salads; tea.	0.08
	*Micromeria myrtifolia* Boiss. and Hohen. (Sulaiman 29)	*Zoufa*	Aerial part	Tea; added to yerba mate.	0.98
	*Origanum syriacum* L. (Sulaiman 24)	*Zauba’*	Leaves	Main ingredient of *Zaatar* (dried and grinded and then *R.* *coriaria* and sesame are added); condiment with salad and strained yogurt; added to yerba mate.	1
	*Salvia officinalis* L. (NC)	*Quaisineh,* *Qasa’een, Mariamiah*	Leaves	Added to yerba mate.	0.06
	*Teucrium procerum* Boiss. and Blanche. (NC)	*Qentariah*	Aerial part	Added to yerba mate.	0.12
	*Thymus vulgaris* L. (Sulaiman 25)	*Za’atar* *barri*	Leaves	Fresh as a condiment for salads; dried and grinded as spices for the traditional cheese *Shanklish*; added to *Zaatar*.	0.72
	*Ziziphora* sp. (NC)	*Qernaieh*	Young aerial part	Steamed with *Sleeq*; added to yerba mate.	0.1
Lauraceae	*Laurus nobilis* L. (Sulaiman 33)	*Ghar*	Leaves	Flavoring of meat.	0.2
Fabaceae	*Cercis siliquastrum* L. (Sulaiman 40)	*Shajreeq*	Flowers	Snack	0.12
	*Melilotus officinalis* (L.) Pall. (Sulaiman 47)	*Handkouq*	Young aerial part	Steamed with *Sleeq*.	0.12
	*Lathyrus sativus* L. (NC)	*Jelbaneh*	Fruits	Snack	0.06
	*Trifolium pratense* L. (Sulaiman 35)	*Neffleh*	Flowers	Added to yerba mate.	0.06
	*Trigonella foenum-graecum* L. (Sulaiman 41)	*Helbeh*	Fruits	Added to yerba mate.	0.28
Malvaceae	*Corchorus olitorius* L. (NC)	*Mlokhiah*	Leaves	Soup: with meat, oil, and lemon added on taste.	0.08
	*Malva sylvestris* L. (Sulaiman 23)	*Khebbaizeh*	Young aerial part	*Marshusheh*: steamed with onion, olive oil and a bit of bulgur).	0.84
Myrtaceae	*Myrtus communis* L. (Sulaiman 7)	*Hinblas*	Fruits	Snack	0.14
Plantaginaceae	*Plantago lanceolata* L. (NC)	*Lsan* *alhamal*	Young aerial part	Steamed with *Sleeq.*	0.1
Polygonaceae	*Rumex acetosa* L. (Sulaiman 38)	*Hmmaidah*	Young aerial part	Steamed with *Sleeq;* boiled as a soup with A. *maculatum;* rice stuffing; salad.	0.74
Portulacaceae	*Portulaca oleracea* L. (NC)	*Beqaileh* *barriah*	Young aerial part	Salad	0.28
Primulaceae	*Cyclamen libanoticum* Hildebr. (NC)	*Doghnain*	Leaves	Rice stuffing	0.4
	*Cyclamen persicum* Mill. (Sulaiman 15)	*Doghnain*	Leaves	Rice stuffing	0.4
Ranunculaceae	*Anemone coronaria* L. (Sulaiman 1)	*Shaqaeq* *alnoa’man*	Young aerial part	Steamed with *Sleeq.*	0.4
	*Ficaria verna* Huds. (Sulaiman 34)	*Mghayriqah*	Young aerial part	Steamed with *Sleeq.*	0.06
Rosaceae	*Crataegus azarolus* L. (NC)	*Za’arour asfar*	Fruits, Flowers	Snack	0.38
	*Crataegus monogyna* Jacq. (Sulaiman 5)	*Za’arour ahmar*	Fruits, Flowers	Snack	0.38
	*Pyrus syriaca* Boiss. (Sulaiman 13)	*Mrab* *barri*	Fruits	Fruit	0.08
	*Rubus sanctus* Schreb. (Sulaiman 6)	*Dees*	Fruits	Snack	0.08
Urticaceae	*Urtica dioica* L. (NC)	*Qerras*	Young aerial part	Steamed with *Sleeq.*	0.12

^1^: N = number of informants; ^2^: NC = not collected.

**Table 2:** Main nutritional benefits of WFPs, which are among the most reported in the current study.

Species Name	Part Used	Main Nutritional Benefits
*Origanum syriacum*	Leaves	Antioxidant [52].
*Rhus coriaria*	Fruits	Good source of phenolics, anthocyanins, organic acids (e.g., malic acid, citric acid and ascorbic acid) and carbohydrates [44].
*Eryngium creticum*	Young aerial part	Rich in antioxidant [49].
*Cichorium intybus*	Young aerial part	Rich in K, P, and vitamin C. It has anti-inflammatory and anti-diabetic activity [45].
*Allium ampeloprasum*	Young aerial part and bulb	Good source of fiber and zinc [48].
*Gundelia tournefortii*	Leaves midrib and underground stem	Good source for minerals K, Ca, P, Na, Fe, Mg, and Zn; as well as in vitamin E [50].
*Scandix pecten-veneris*	Young aerial part	Highly rich in Fe; a significant source of Zn [53].
*Malva sylvestris*	Young aerial part	Rich in Ca, Mg, and K [51].
*Nasturtium officinale*	Young aerial part	A good source of K and Fe [53].
*Rumex acetosa*	Young aerial part	Rich in proteins [47,54].
*Thymus vulgaris*	Leaves	Has antioxidative, anti-inflammatory, antibacterial and antifungal activity [46].

## Data Availability

The data presented in this study are available on a reasonable request from the corresponding author.
